# Assessment of predictive value of pre- and postoperative urethral pressure profiles for long-term continence in female dogs with ectopic ureters: a preliminary study

**DOI:** 10.1186/s13028-025-00848-z

**Published:** 2026-02-02

**Authors:** Pierre Langer, Annick Hamaide, Stéphanie Noël

**Affiliations:** Department of Companion Animal Clinical Sciences, Faculty of Veterinary Medicine, University of Liège, Liège, Belgium

**Keywords:** Canine, Incontinence, Urodynamics

## Abstract

**Background:**

Ectopic ureters (EU) are the main cause of urinary incontinence in juvenile dogs with a continence rate ranging from 22 to 72% reported after surgical correction. The objective of this retrospective preliminary study was to evaluate the usefulness of pre- and postoperative urethral pressure profiles (UPP) in predicting long-term continence in dogs with EU.

**Results:**

UPP were performed in 16 female dogs prior to surgical correction of EU, as well as postoperatively, between 2012 and 2022. Urodynamic parameters included maximal urethral pressure (MUP), maximal urethral closure pressure (MUCP), anatomical profile length (APL), functional profile length (FPL) and integrated pressure (IP). A continence score (CS), defined as 1 = incontinent, 2 = continent with sporadic episodes of incontinence, 3 = continent, was given at the time of pre- and postoperative UPP. Neoureterostomy with dissection of the intramural portion (23 EU) or neoureterocystostomy (3 extramural EU) were performed. Seven dogs were neutered before or during surgical correction. Median preoperative CS was 1 (min 1, max 2). All dogs were continent with a CS of 3 in the immediate postoperative period. Long-term median follow-up time was 24 [8.5–42] months. Recurrence of incontinence occurred in 9 dogs (56.3%). Median time duration without recurrence was 5 months. In the 16 dogs, postoperative FPL values (median 70.5 [56-82.5] mm) were significantly increased compared to preoperative values (median 56.5 [41-72.3] mm) (*P* = 0.034). In the group of 7 dogs without recurrence of incontinence, IP increased significantly from a preoperative median value of 102 [19–171] cm.cmH2O to a postoperative median value of 132 [67–225] cm.cmH2O (*P* = 0.016). In dogs without recurrence, ranges of variation between pre- and postoperative MUP and IP values, as well as postoperative MUCP values (median 47.3 [24.5–52] cmH2O, *P* = 0.026) were significantly higher (*P* = 0.017 and *P* = 0.039 respectively). Recurrence hazard of incontinence was neither significantly associated with age, breed, preoperative urodynamic measurements, CS, neutering, or the type of EU.

**Conclusions:**

In our population, preoperative UPP could not be considered as a diagnostic procedure predictive for incontinence recurrence after surgical correction of EU. Our urodynamic findings support potential improvement in urethral tone in female dogs without recurrence of incontinence.

## Background

Ectopic ureters (EU) are the primary cause of urinary incontinence in juvenile dogs [1, 2, 3] while the second most common cause is congenital urethral sphincter mechanism incompetency (CUSMI), which has been described in medium to large breed dogs [1].

Surgical treatment of EU consists of neoureterocystostomy or neoureterostomy [4]. Cystoscopic-guided laser ablation is also described [5, 6]. Postoperative continence rate after surgical correction of EU without additional treatment ranges from 22 to 72% [7–14]. In one study, recurrence of urinary incontinence after a phase of complete continence was observed in 32% of dogs after surgical correction of EU [15]. The long-term continence rate improved with medical treatment, and neutering was not associated with an increased risk ofincontinence recurrence [15]. In a more recent study, recurrence of incontinence was described in up to 25% of dogs after surgical correction of EU [16]. Several hypotheses for postoperative persistent or recurrent incontinence have been proposed, such as urinary tract infection (UTI), recanalization of the ureter remnant, disturbed urethral closure due to residual intramural EU, CUSMI, hypoplastic bladder, vestibulovaginal stenosis, neurogenic abnormalities, or inadequate surgery [2, 7, 8, 10, 13, 17].

While previous studies reported that the type, the side, the number of EU, the presence of UTI, the type of surgical technique and the presence of preoperative hydroureter did not influence postoperative continence in affected dogs [8–10], a recent study showed that preoperative dilation of the ureter which may result from vesico-urethral sphincter contractions on the ectopic part of the ureter was associated with a higher chance of continence after surgery [18]. Moreover, it has been shown in female Golden Retrievers that presence of historical UTI prior to EU correction was negatively correlated with postoperative urinary continence whilst preoperative ureteral dilatation was positively correlated with likelihood of short-term urinary continence [19].

Urodynamic investigation, including UPP and cystometry, is described to assess vesico-urethral function in dogs [20]. Indeed, UPP allows a quantitative description of the urethral pressure from the bladder neck to the urethral meatus, and is useful to characterize USMI in dogs [20–23]. Urodynamics has been used to evaluate vesico-urethral function in dogs with EU. In 9 dogs with EU, Lane and collaborators showed that 67% of those dogs had preoperative urodynamic characteristics of USMI and 44% of them had a small bladder capacity [10]. In another study, a low urethral pressure was recorded in 4 out of 4 dogs with EU but UPP was performed before surgical correction in 2 dogs, and several years postoperatively in the remaining dogs [11]. Potential benefit of preoperative urodynamic examination has been recently suggested for predicting postoperative urinary continence as dogs affected by EU condition may show concurrent USMI [18, 19].

A study evaluating values of pre- and postoperative urodynamic parameters related to continence status in female dogs with EU condition is currently lacking in the literature.

The objective of this preliminary study was to evaluate the usefulness of pre- and postoperative UPP in predicting long-term continence after surgical correction of EU in female dogs.

## Methods

### Inclusion criteria

Medical records of female dogs that underwent surgical correction of EU as well as pre- and postoperative UPP, between 2012 and 2022, were reviewed. Preoperatively, urine culture was carried out if UTI was suspected based on clinical history.

UPP were not performed in dogs with diagnosed UTI based on positive urine culture [3]. Data included breed (assigned to one of the following groups based on the size of the dog: giant, large, medium and small breed dogs), neuter status, urodynamic recordings, type of surgery (neoureterocystostomy, neoureterostomy), and continence status. Follow-up information was obtained by patient recheck evaluation and owner telephone interview. Female dogs diagnosed with a pelvic bladder condition, defined as a bladder with > 5% of its length located in the pelvis based on cystography [24], were not included in the study.

### Diagnostic procedures

Complete blood count, biochemistry, urinalysis (including cytology), as well as urine culture were performed prior to imaging procedures.

Diagnostic imaging procedures included abdominal ultrasonography, intravenous pyelogram combined with pneumocystography and vagino-urethrography. If needed, computed tomography and cystoscopy were also performed.

### Urethral pressure profiles

Urodynamic examinations were performed under IV continuous rate of propofol anesthesia as previously described [25], immediately before surgery and approximately 1 month after surgical correction. To summarize, when dogs were under a stable plane of anesthesia, they were placed in right lateral recumbency. Depending on the size of the dogs, a sterile 6Fr double lumen perfusion catheter with 1 side-hole or a sterile 8Fr or 10Fr polyvinylchloride triple lumen perfusion catheter with 2 side-holes (Urodynamic, Coloplast©, Belgium) was inserted via the urethra within the bladder. Sterile saline solution was infused via the perfusion catheter, which was clamped on a mechanical puller arm that withdraws the catheter at a speed of 1 mm/sec. Three successive UPP measurements were recorded with a computer-based urodynamic system (Libra + Medical Measurement Systems, Benetec©, Belgium).

**Fig. 1:**
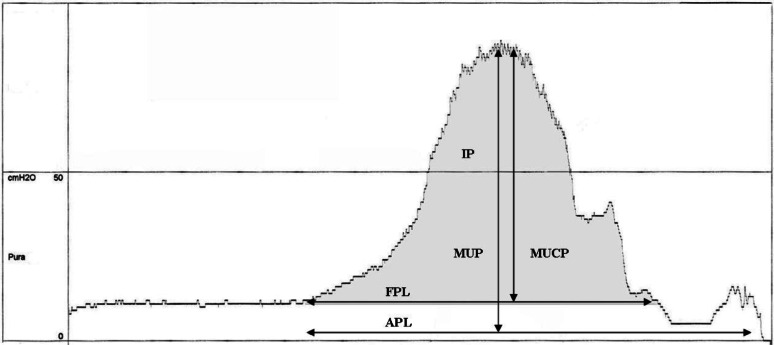
Graphic representation of a urethral pressure profile in a healthy continent female dog. Pura, urethral pressure (cmH2O); MUP, maximal urethral pressure (cmH20); MUCP, maximal urethral closure pressure (cmH2O); FPL, functional profile length (mm); IP, integrated pressure (functional profile area, area under the FPL curve) (cm.cmH2O); APL, anatomical profile length (mm)

Urodynamic parameters included maximal urethral pressure (MUP), maximal urethral closure pressure (MUCP, the difference between maximal urethral pressure and bladder pressure), anatomical profile length (APL, the distance between the point in the urethra where intra-urethral pressure exceeds the total bladder pressure and the point where the intra-urethral pressure decreases to atmospheric pressure), functional profile length (FPL, the distance between the point in the urethra where intra-urethral pressure exceeds bladder pressure and the point where either a pressure plateau is observed or where the intra-urethral pressure drops below bladder pressure) and integrated pressure (IP, the area under the FPL curve = MUCP*FPL) (Fig. 1) [25]. Definitions of these variables are in accordance with those accepted by the International Continence Society [26].

### Surgical procedures

In case of extramural EU, a neoureterocystostomy was performed. In case of intramural EU, a neoureterostomy with dissection of the intramural portion of the EU was performed [27]. Ovariectomy was performed during the same procedure in some bitches depending on owner’s preference.

### Data analysis

A continence score previously described [15, 28] (1 = incontinent, 2 = continent with sporadic episodes of incontinence, 3 = continent) was given at the time of pre- and postoperative UPP recordings. Recurrence of urinary incontinence was assessed during the postoperative recheck or was obtained from owner questionnaire. Data were analyzed based on incontinence recurrence status and sterilization status.

Categorical variables were described using numbers and percent whilst continuous variables were described using median and interquartile range [Q1-Q3] or extreme values (Min, Max). Influence of timing of UPP (preoperative versus postoperative UPP), age, breed, neutering, and recurrence of urinary incontinence, on the values of urodynamic parameters were analyzed. Pre- and postoperative median values of urodynamic parameters and CS were compared using a Wilcoxon test. Median values of urodynamic parameters and CS in the two groups of dogs (with or without postoperative recurrence) were compared using a Kruskal-Wallis test. Cox regression (upon timing) was performed to evaluate if the recurrence hazard was associated with preoperative UPP values, age, breed, type of EU and sterilization status. Statistical analysis was performed using SAS software version 9.4 (SAS Institute, Cary NC, USA). *P* values < 0.05 were considered significant.

## Results

Sixteen female dogs were included in the study. Breed types included 1 giant, 9 large, 5 medium and 1 small type. The median age at the time of preoperative UPP was 6.5 months [3.75-11] and the median age at the time of postoperative UPP was 8.75 months [5.25–13.8]. Six dogs had unilateral and 10 had bilateral EU. Twenty-three intramural EU were corrected by neoureterostomy with dissection of the intramural portion. Three extramural EU were corrected by neoureterocystostomy. Seven dogs were neutered before or during surgical correction and 9 dogs were left intact. Based on clinical history and the age of dogs at the time of being operated, 10/16 had not had their first estrous cycle yet and 6/16 dogs had already had it. Nine out of 16 dogs had a preoperative urine culture performed and 6/9 were positive. Those six dogs received targeted antibiotherapy for three weeks before preoperative UPP and surgery completion. Among the seven dogs who did not have urine culture performed, 6/7 had urinalysis (cystocentesis) revealing negative urine sediments for bacteria and cells at cytology. One out of 16 dogs did not have urinalysis performed but her clinical history did not reveal clinical signs compatible with UTI such as hematuria or pollakiuria. Before the postoperative UPP’s, 4/16 dogs had a urine culture performed which was positive for 3 dogs and treated with a targeted antibiotherapy. Among the 12 dogs who did not have urine culture performed, 11/12 had urinalysis (cystocentesis) revealing negative urine sediments for bacteria and cells at cytology. One out of 16 dogs did not have urinalysis performed but her clinical history did not reveal clinical signs compatible with UTI such as hematuria or pollakiuria.

Data regarding pre- and postoperative CS and the time to recurrence of incontinence are detailed in Table 1. Fifteen dogs had a preoperative CS of 1 with continuous dribbling of urine and one dog had a preoperative CS of 2 being incontinent only during episodes of cystitis. At the time of discharge, 48 h postoperatively, all dogs had a CS of 3 and were fully continent.

**Table 1:**
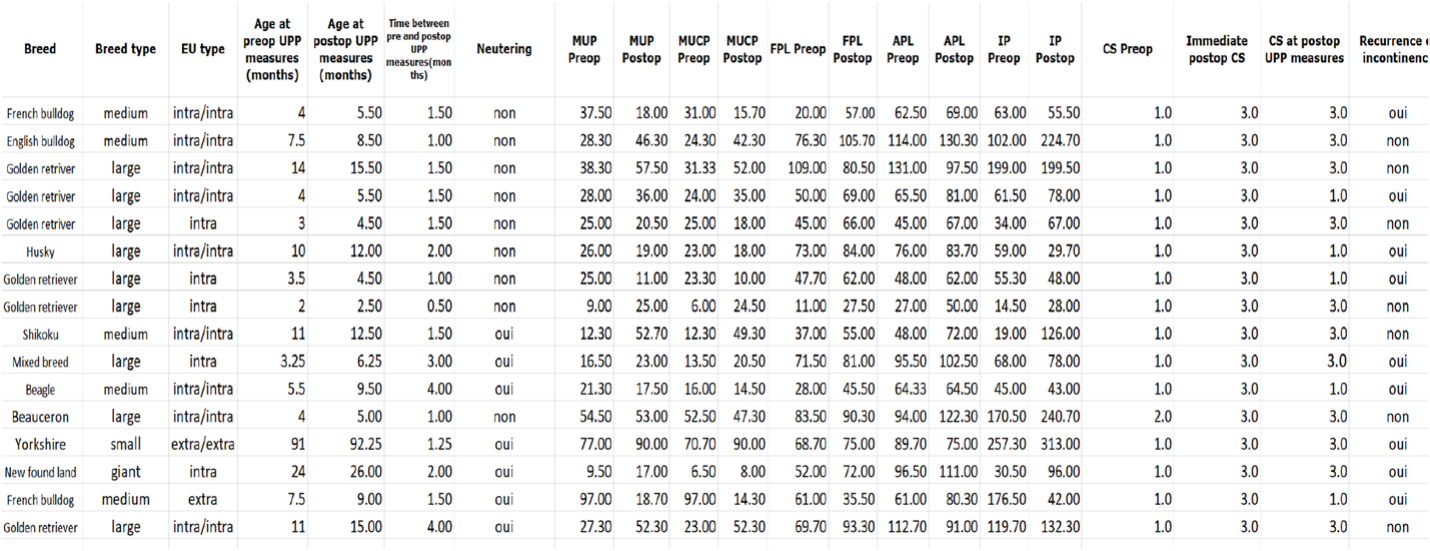
Breed, size, sex, EU type, UPP values, neutering status, recurrence, CS and follow-up data

UPP: urethral pressure profile; UPP values are described as mean of three successive measurements for each parameter; EU: ectopic ureter; MUP: maximum urethral pressure (cmH2O); MUCP: maximum urethral closure pressure (cmH2O); APL: anatomical profile length (mm); FPL: functional profile length (mm); IP: integrated pressure (cm.cmH2O); CS: continence score

### Time duration without recurrence of incontinence

At the time of postoperative UPP (median 1.5 months [1.13-2]), recurrence of incontinence was observed in 5 dogs. However, CS of the 16 dogs improved significantly from a median score of 1 to a median score of 3 (*P* = 0.001).

Long-term median follow-up time was 24 [8.5–42] months, with a maximum of 110 months.

During long-term follow-up, recurrence of incontinence developed in 4 additional dogs and the median time duration without recurrence was 5 months. The 7 dogs without recurrence of incontinence had a median follow-up time of 30 [10–51] months.

**Fig. 2:**
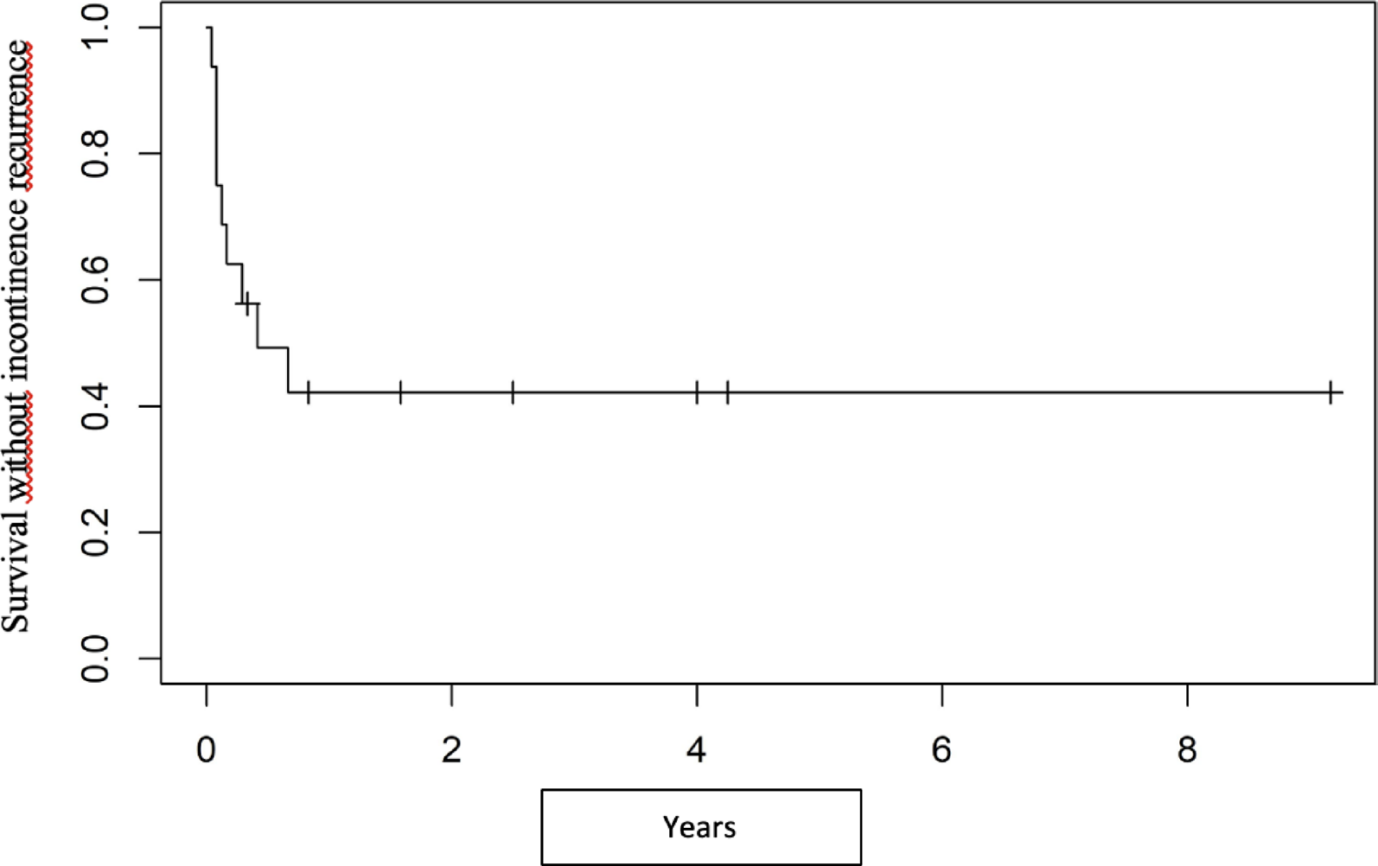
Kaplan Meier curve of survival time without incontinency recurrence

Probability of postoperative continence was 75%, 62.5% and 49.2% at 1, 2 and 6 months respectively (Fig. 2). In total, recurrence of incontinence occurred in 9 dogs (56.3%). In these dogs, urinary leakage occurred in recumbent position or during increased activity.

### Overall comparison of the pre- and postoperative urodynamic values

Urodynamic values are detailed in Table 1.

Overall comparison of pre- and postoperative values of urodynamic parameters revealed that MUP and MUCP values remained stable. Median preoperative and postoperative MUP values were 26.7 [18.9–37.9] and 24 [18.4–52.5] cmH_2_O respectively with no significant difference (*P* = 0.35). Median preoperative and postoperative MUCP values were 23.7 [14.8–31.2] and 22.5 [15.1–48.3] cmH_2_O respectively with no significant difference (*P* = 0.29). Postoperative values of FPL (median 70.5 [56-82.5]mm) were significantly increased compared to preoperative values (median 56.5 [41-72.3] mm) (*P* = 0.034). APL and IP values tended to increase postoperatively but not significantly. APL increased from a median value of 70.8 [54.5–96] mm to 80.7[68–100] mm (*P* = 0.08). IP increased from a median value of 62.3 [39.5–145]cm.cmH2O to 78 [45.5–166] cm.cmH2O (*P* = 0.08).

### Comparison of urodynamic values and CS between the 2 groups of dogs (with or without recurrence of incontinence)

In the group of 9 dogs with recurrence of incontinence, no significant changes were observed between the pre- and postoperative urodynamic values, although a nonsignificant trend to increase was observed for FPL and APL values. Following an immediate postoperative CS of 3, and as recurrence occurred, CS for those 9 dogs remained stable with a score of 1.

In the group of 7 dogs without recurrence of incontinence, IP increased significantly from a preoperative median value of 102 [19–171] cm.cmH2O to a postoperative median value of 132 [67–225] cm.cmH2O (*P* = 0.016). MUP and MUCP values tended to increase but not significantly. FPL and APL values remained stable. CS significantly increased from 1 to 3 (*P* = 0.016).

Comparison of pre- and postoperative values of urodynamic parameters between the 2 groups of dogs showed that preoperative UPP values were homogenous and comparable between the 2 groups. When comparing the ranges of variation between pre- and postoperative MUP and IP values, the range increase between pre- and postoperative MUP and IP values was significantly higher in dogs without recurrence (*P* = 0.017 and *P* = 0.039 respectively). Postoperative MUCP values were significantly higher in dogs without recurrence (median 47.3 [24.5–52] cmH2O, *P* = 0.026).

### Comparison of urodynamic values and CS based on sterilisation status

Four out of the 9 intact dogs (57.1%) and 5 out of the 7 neutered dogs (71.4%) showed recurrence of incontinence. However, risk of recurrence was not associated with sterilization (OR = 3.13, *P* = 0.29).

In the group of intact dogs, MUP, MUCP, APL and IP values remained stable at the postoperative UPP evaluation. FPL values showed a trend to increase with a median difference of 16.5 [11–21] mm (*P* = 0.074). CS improved significantly from 1 to 3 (*P* = 0.031).

In the group of neutered dogs, none of the urodynamic parameters significantly changed over time. CS showed a trend to increase (*P* = 0.063).

Between the two groups, preoperative urodynamic parameters were homogenous. No significant difference was observed in values urodynamic parameters or in CS over time.

### Assessment of risk of recurrence

Cox regression revealed that risk of recurrence was neither significantly associated with age, breed, preoperative urodynamic measurements, CS, neutering or the type of EU (Table 2).

**Table 2:** Cox regression for preoperative measurements, surgery and immediate postoperative period

Variable	*N*	Event	HR	HR lower limit	HR upper limit	*P*-value
Large/Giant VS Small/medium breed	16	9	0.666	0.178	2.489	0.55
Age at preop urodynamics measures (months)	16	9	1.061	0.557	2.018	0.86
Preop MUP (cmH2O)	16	9	1.583	0.560	4.479	0.39
Preop MUCP (cmH20)	16	9	1.439	0.562	3.687	0.45
Preop FPL (mm)	16	9	0.993	0.969	1.017	0.55
Preop APL (mm)	16	9	0.993	0.973	1.014	0.52
Preop IP (cm.cm H2O)	16	9	1.169	0.560	2.440	0.68
Preop CS	16	9	0.528	0.026	10.818	0.68
Intra VS extra EU	16	9	3.907	0.821	18.596	0.087
Unilateral VS bilateral EU	16	9	0.680	0.182	2.542	0.57
Neutering (Y/N)	16	9	1.913	0.510	7.169	0.34

N: Number of dogs; Event: number of dogs who underwent recurrence of incontinence; EU: ectopic ureter; HR: hazard ratio; MUP: maximum urethral pressure; MUCP: maximum urethral closure pressure; APL: anatomical profile length; FPL: functional profile length; IP: integrated pressure; CS: continence score

## Discussion

In this preliminary study, we report that preoperative urodynamic measurements, age, breed, CS, neutering or the type of EU are not predictive for long-term continence after surgical correction of EU.

Recurrence hazard associated to the postoperative UPP measurements could not be assessed as the period between pre- and postoperative measurements was sometimes longer compared to the period between preoperative measurements and the timing of recurrence. Nonetheless, urodynamic values increased or tended to increase in dogs without recurrence and remained stable in dogs with recurrence. Indeed, while values of preoperative urodynamic parameters were not significantly different between dogs with or without recurrence of incontinence after surgical correction of EU, ranges of variation in postoperative median MUP and IP values, as well as in postoperative MUCP values, were significantly higher in dogs without recurrence. The urodynamic values in those dogs seem to follow the same trend as described in a previous study in normal healthy female dogs [29]. Indeed, in sexually immature healthy female Beagle dogs, MUCP, IP and FPL values increase significantly at 7 months of age and APL at 8.5 months of age [29]. In the present study, our median preoperative and postoperative age of the dogs at the time of UPPs were 6.5 and 8.75 months, respectively, which are close from 7 to 8.5 months in the Beagles population. Therefore, the results of our study may reflect a weaker urethral tone in dogs developing recurrence of incontinence.

Considering that EU can be associated with USMI, UPP could be an appropriate tool to diagnose USMI and help predict postoperative long-term continence in dogs affected with EU. Indeed, in dogs with USMI, a weak urethral tone is objectified by low MUCP and FPL values [21–23].

Previously, urodynamics values were recorded in normal intact prepubertal and young adult Beagle bitches under the same anesthetic conditions as in this present study, and a mean MUCP value of 62 cmH_2_O at 9 months of age (before the first estrous cycle), 70 cmH_2_O during their late anestrous phase of their first estrous cycle, a mean MUCP value of 80 cmH_2_O during their late anestrous phase of their second estrous cycle were reported [29]. In that study, an increase in urethral resistance was described around 7 months of age in prepubertal female Beagle dogs. Increase of urethral lengths and pressures were reported until the second estrous cycle in femaleBeagle dogs [29]. However, in this previous study, age was the main factor affecting the UPP values (around 7 months of age whilst the dogs were still in prepubertal period) and not the actual occurrence of estrous cycle. In our population, the median preoperative UPP age was 6.5 months which is close from the 7 months in the Beagle population suggesting that age may play a role in development of recurrence of incontinence.

In the present study, when comparing groups of dogs with and without recurrence, median postoperative MUCP values of dogs without recurrence were significantly higher than preoperative values, with those postoperative values being close to the values reported by Noël and collaborators in normal continent bitches. In another study, preoperative UPP in 9 dogs with EU were evaluated [10]. A MUCP value higher than 19 cm H20 was considered predictive of postoperative continence and these predicted outcomes were consistent with actual outcomes in 8 of 9 dogs. They also reported that the functional profile area, corresponding to IP, was significantly lower in dogs remaining incontinent after surgery [10]. As anesthesia protocols used by Lane and collaborators were different from the protocols used in this study, comparisons cannot be made with our current results. Further prospective investigations on the reliability of urodynamic values on a larger homogenous population of dogs are needed in order to confirm these preliminary results.

The significant increase in postoperative FPL values may be linked to improvement in urethral function. In the present study, all 16 dogs were fully continent at the time of discharge and 11 were still continent at the time of postoperative UPP, emphasizing the fact that resection of the intramural portion of the EU probably did not alter urethral function in the immediate postoperative period. Among the remaining five dogs with recurrence of incontinence postoperatively, four showed low postoperative urethral pressure values compatible with a diagnosis of USMI. Despite an excellent immediate postoperative continence rate, recurrence of urinary incontinence was observed in 9/16 (56%) of the dogs. In those dogs, the pattern of urine leakage was different with occurrence of incontinence in recumbent position or during increased activity, while continuous dribbling of urine was observed before surgery. This high rate of recurrence is difficult to interpret given the small number of dogs included in this study, but some hypotheses can be suggested. During urethral surgery, urethral tissue manipulation and increased duration of surgery can lead to marked mucosal edema and inflammation [30]. These physiological reactions could act as local bulking agent participating in urinary continence in the immediate postoperative time. In a rat model of urethral injury, the different phases of urethral wound healing were longer than dermal healing and involved a vast majority of the peri-urethral tissue [31]. In men, after robot-assisted radical prostatectomy, peri-urethral inflammation negatively impacts continence status as it induces urethral fibrosis and increases collagen content leading to lower urinary tract symptoms [32]. Development of postoperative urethral fibrosis after resection of intramural EU in dogs in our study could affect continence mechanisms; however, in a previous study, no significant differences in terms of postoperative continence could be identified between ligation and resection techniques in dogs [9]. Histological studies of the urethral wall would be warranted to verify this hypothesis, but urethral biopsies are invasive procedures in dogs. Advanced imaging techniques such as MRI could be useful to assess evolution of the urethral wall remodeling after EU surgery. More recently, it was reported in three different studies that EU cystoscopic-guided laser ablation was associated with various rates of recurrence of incontinence compared to surgical correction [16, 19, 33]. A prospective study comparing postoperative UPP values in dogs that underwent EU correction with the two different techniques may potentially help with assessing the urethral function and a potential USMI condition in those dogs, assuming their age is comparable.

One study has suggested that a favorable postoperative continence rate (72%) after surgical correction of EU could be partly explained by the high proportion of entire female dogs included [2]. Indeed, USMI can occur in 3.1% [34] to 20% [35, 36] of female dogs approximately 3 years after spaying, and 90% of bitches with USMI are spayed [1, 37]. Neutering is associated with a decrease in bladder and urethral smooth muscle, and an increased proportion of collagen [38–40] which could impair the functional integrity of the lower urinary tract. However, previous studies reported that neutering (either before or during surgical correction of EU) and age at neutering were not associated with an increased recurrence hazard of incontinence [15, 18].

In the present study, neutering was performed at the time of surgical correction of EU due to the congenital nature of this disease depending on the owner’s preference. In our population of dogs, neutering was not found to influence the recurrence risk of incontinence. The time frame between the correction of EU and the recurrence of postoperative incontinence in our group of spayed female dogs was shorter than the time frame reported between neutering and the onset of incontinence in dogs developing acquired USMI. This makes unlikely the hypothesis that neutering and development of acquired USMI would be the cause of the relapse of incontinence in the dogs of the present study. Therefore, it further supports the hypothesis of a potential pre-existing congenital form of USMI in those dogs affected with EU who showed recurrence of incontinence after the surgical correction of their EU.

Given the retrospective nature of this preliminary study, the major limitations include the variability of the population and the low number of dogs. Indeed, we included dogs with different ureteral anatomical variations (intra or extramural EU) and different surgical procedures (neoureterocystostomy, neoureterostomy, ovariectomy). Further studies on a larger population of dogs are needed to allow distribution in larger homogenous groups. However, it must be noted that previous studies showed no significant effect of neutering nor surgical technique on recurrence of urinary incontinence after surgical correction of EU [2, 15, 16, 18]. Another limitation is the heterogeneity in age and breed of the dogs although the majority of dogs were less than 1 year of age and were from large breeds. We included only female dogs because male dogs show different urodynamic characteristics. Limitations with urodynamic examinations are well described. Indeed, despite the usefulness of urodynamic examination in the management of urethral dysfunction, results obtained with this technique can show variability and lack of repeatability. Several factors can influence UPP such as animal position [41], orientation of the transducer within the urethra [41], type of catheter [42, 43], type of sedatives [20, 44, 45], catheter withdrawal rate [46], fluctuations of the abdominal pressure [46], degree of bladder repletion [43, 46] and hormonal status of the dog [29, 47]. To avoid these artefacts, UPP in this study were performed under the recommended standardized conditions [26] and under propofol sedation. Propofol was used to induce a light plane of anesthesia as previously described [25] that allows limited artefacts present in awake dogs and good repeatability of the measurements without major impact of sedation on urodynamic values. Finally, the continence score used in this study could be considered as a limitation as subtle differences in outcome could have been missed, but due the retrospective nature of this study, a more sensitive score could not be used. Furthermore, the owners facing recurrence of urinary incontinence, even if improved, are still inconvenienced by the incontinence of their pet.

## Conclusions

In this population of female dogs, preoperative UPP could not be considered as a diagnostic procedure predictive forrecurrence of urinary incontinence after surgical correction of EU. Improvement in urethral tone might be suggested in dogs without incontinence recurrence as supported by our urodynamic findings. Further prospective studies involving a larger number of dogs are warranted to confirm these preliminary results.

## Data Availability

The data sets used and/or analysed during the current study are available from the corresponding author on reasonable request.

## Abbreviations

APL: anatomical profile length
CS: continence score
CUSMI: congenital urethral sphincter mechanism incompetency
EU: ectopic ureters
FPL: functional profile length
IP: integrated pressure
MUCP: maximal urethral closure pressure
MUP: maximum urethral pressure
UPP: urodynamic pressure profiles
UTI: urinary tract infection
